# Response to: Comment on “Isolated perineal plaque as the initial presentation of pemphigus vulgaris”

**DOI:** 10.1016/j.jdcr.2026.02.036

**Published:** 2026-02-25

**Authors:** Aster Workineh, Gauri Panse, Mary Tomayko, Alicia J. Little

**Affiliations:** a Yale School of Medicine, New Haven, Connecticut; b Department of Dermatology, Yale School of Medicine, New Haven, Connecticut

**Keywords:** desmoglein 3, isolated pemphigus, localized pemphigus, mucosal-dominant pemphigus, pemphigus, pemphigus vulgaris, perineal plaque

*To the Editor:* We thank Dr Morgaonkar for their thoughtful comments and agree that limited reports of vulvovaginal or perineal-predominant pemphigus vulgaris may reflect underrecognition, in part due to patient-level barriers to care-seeking. In a survey of 177 women with vulvar lichen sclerosis, the most common vulvar dermatosis in postmenopausal women, 40% reported hiding symptoms and described the condition as private.^1^ A primary care review likewise noted cultural and social taboos delaying reporting, exacerbated by embarrassment and fear of judgment when symptoms were minimized during initial encounters.^2^

Anogenital lesions mimicking sexually transmitted infections may further deter disclosure, as noted by Dr Morgaonkar. Although delayed referral in our case stemmed from a minimally symptomatic perineal erosion with partial steroid response,^3^ other acantholytic disorders in this anatomic region can mimic infections. In the largest anogenital papular acantholytic dyskeratosis case series, diagnostic delays reached 40 years, with lesions initially misattributed to genital warts.^4^

Underrecognition may be particularly pronounced in older women because routine screening pelvic examinations are not recommended for asymptomatic patients, reducing chances for incidental detection of anogenital dermatoses.^5^

Normalizing anogenital health discussions in routine care and incorporating screening questions may help elicit concerns patients might not readily volunteer. Coupled with high index of suspicion for pemphigus in persistent or treatment-refractory anogenital dermatoses, these strategies may support timely referral and earlier recognition of blistering and other acantholytic dermatoses in this anatomic region. Large multidisciplinary studies, clinician education, and destigmatization efforts are needed to determine true prevalence of anogenital pemphigus vulgaris.

## Conflicts of interest

None disclosed.

## References

[1] Rees S., Arnold S., Parsons H., Hillman S. (2025). Vulval lichen sclerosus in UK general practice: a cross-sectional survey of patient experience. BMJ Open.

[2] Clarke L., Leatherland R., Simpson R.C. (2025). A systematic review of the barriers to diagnosis of vulval lichen sclerosus in primary care. Clin Exp Dermatol.

[3] Workineh A., Panse G., Tomayko M., Little A.J. (2026). Isolated perineal plaque as the initial presentation of pemphigus vulgaris. JAAD Case Rep.

[4] Al-Muriesh M., Abdul-Fattah B., Wang X., Zhao M., Chen S., Huang C. (2016). Papular acantholytic dyskeratosis of the anogenital and genitocrural area: case series and review of the literature. J Cutan Pathol.

[5] (2018). ACOG Committee Opinion No. 754: the utility of and indications for routine pelvic examination. Obstet Gynecol.

